# Synthesis, crystal structure and Hirshfeld surface analysis of 3-[5-(4-chloro­phen­yl)-2-sulfanyl­idene-1,3,4-oxa­diazol-3-yl]-3-(4-methyl­phen­yl)-1-(thio­phen-2-yl)propan-1-one

**DOI:** 10.1107/S2056989026008819

**Published:** 2026-09-03

**Authors:** Shailakshi Jain, Sharatha Kumar, Tuncer Hökelek

**Affiliations:** a Department of Chemistry Yenepoya Institute of Arts Science Commerce and Management Mangaluru Yenepoya University (Deemed to be University) 575013 Karnataka India; b Hacettepe University, Department of Physics, 06800 Beytepe-Ankara, Türkiye; Universität Greifswald, Germany

**Keywords:** 4-oxa­diazol, 4-chloro­phen­yl, crystal structure

## Abstract

The title thio­phene-chalcone derivative, C_22_H_17_ClN_2_O_2_S_2_, contains a planar oxa­diazole, a thio­phene and two phenyl rings. The dihedral angle between the oxa­diazole and thio­phene rings is 27.45 (10)°, and they are oriented with respect to the chloro­phenyl and methyl­phenyl rings at 22.61 (10), 67.07 (8) and 49.16 (10), 87.22 (8)°, respectively, while the phenyl rings are oriented at 51.78 (9)°. In the crystal, π–π stacking inter­actions and C—H⋯π(ring) inter­actions help to consolidate the packing. Hirshfeld surface analysis revealed that the most important contributions to the crystal packing are from H⋯H (31.6%), H⋯C/C⋯H (20.0%), H⋯S/S⋯H (14.2%) and H⋯O/O⋯H (9.5%) inter­actions.

## Abstract 2

The asymmetric unit of the title mol­ecule contains a planar oxa­diazole, a thio­phene and two phenyl rings. In the crystal, π–π stacking inter­actions and C—H⋯π(ring) inter­actions help to consolidate the packing.

## 1. Chemical context

Oxa­diazole is an important structural motif in medicinal chemistry for its role as a pharmacophore and a ligand-binding scaffold (Boström *et al.*, 2012 ▸). Among its four isomeric forms, the 1,3,4-oxa­diazole ring has attracted considerable attention owing to its broad spectrum of biological activities (Salahuddin *et al.*, 2017 ▸; Patel *et al.*, 2014 ▸). Numerous 1,3,4- and 1,2,4-oxa­diazole derivatives have been reported to exhibit anti­bacterial, anti-inflammatory, anti­tubercular, anti-HIV, anti­fungal, cathepsin K inhibitory, mono­amine oxidase inhib­itory, anti­diabetic, tyrosinase inhibitory, anti­oxidant and anti­cancer activities (Palaska *et al.*, 2002 ▸; Kadi *et al.*, 2007 ▸; Alisi *et al.*, 2020 ▸).

In recent years, thio­phene-containing chalcone derivatives have emerged as an important class of compounds because of their extended π-conjugated systems and promising applications in medicinal and materials chemistry (Shalaby *et al.*, 2023 ▸). Adding heterocyclic moieties to the chalcone scaffold is a well-established approach for modulating mol­ecular geometry and physicochemical properties. In this context, the integration of a 1,3,4-oxa­diazole ring with a thio­phene-chalcone framework generates conjugated mol­ecular systems of significant structural and potential biological inter­est. As part of our ongoing research on heterocyclic compounds, the title thio­phene-chalcone derivative containing a 1,3,4-oxa­diazole unit was synthesized and its crystal structure was elucidated to investigate its mol­ecular conformation and inter­molecular inter­actions governing the crystal packing. Herein, we report the mol­ecular and crystal structures together with a Hirshfeld surface (HS) analysis of the title compound.
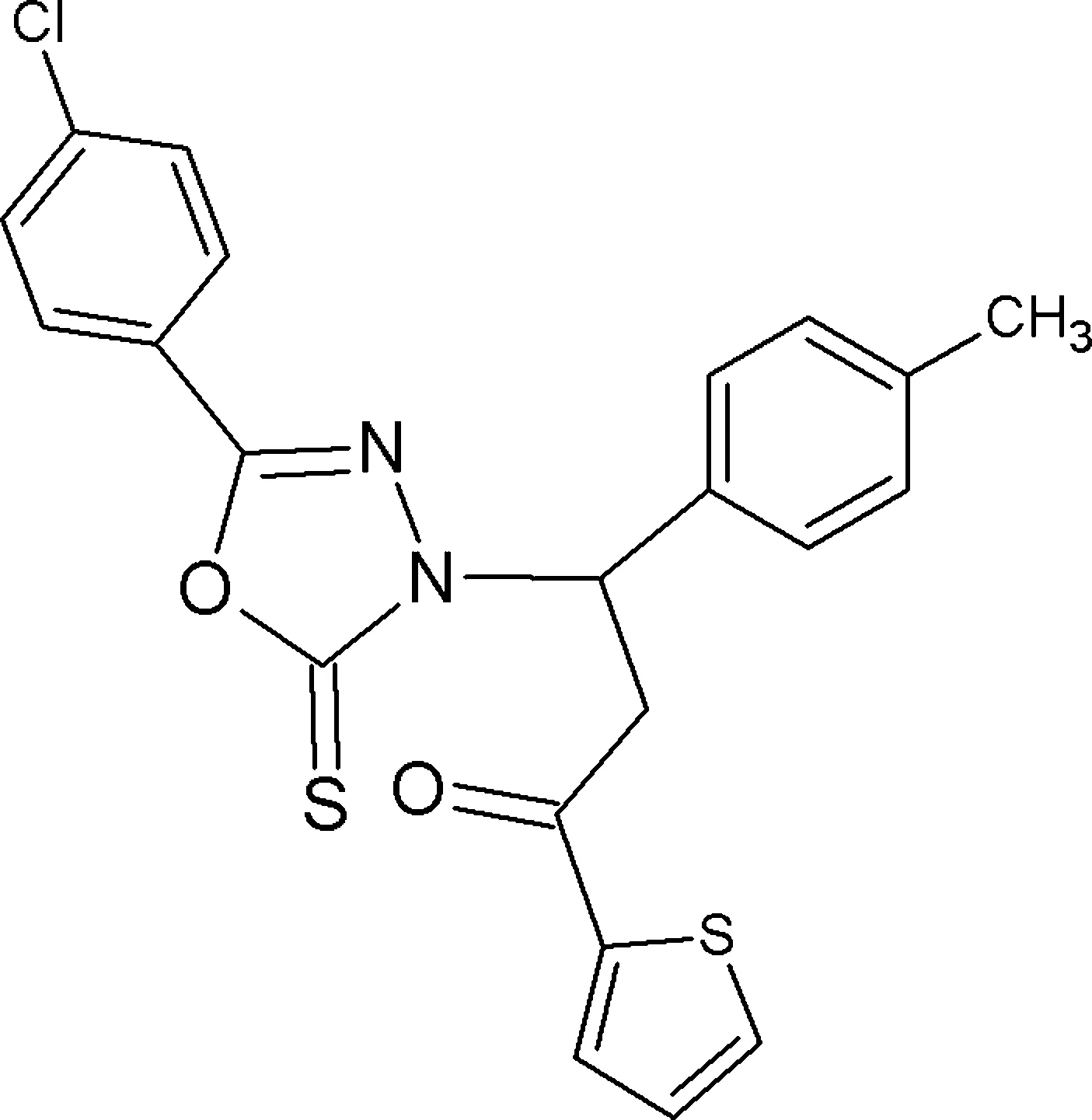


## 2. Structural commentary

**Figure 1:**
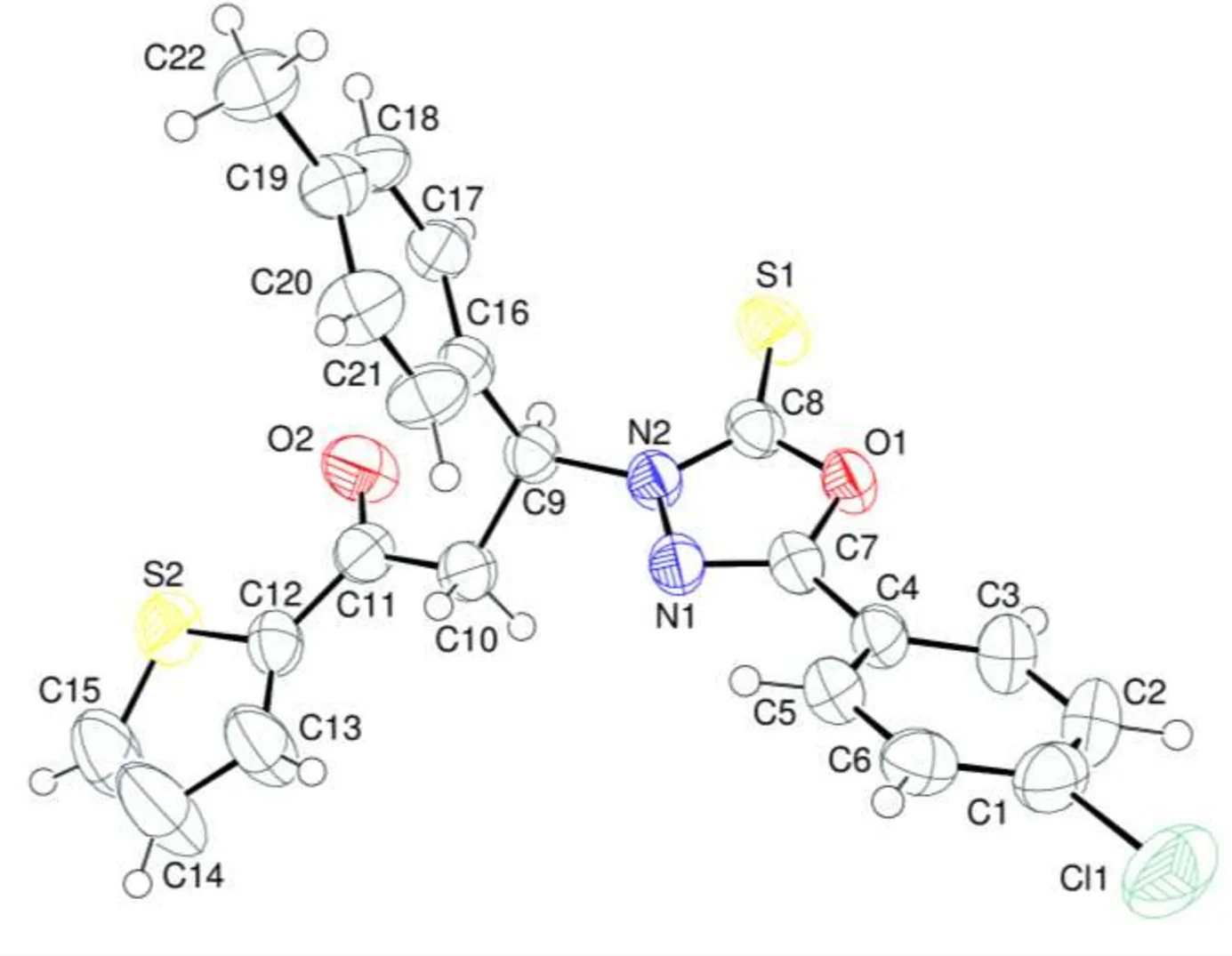
The asymmetric unit with atom-numbering scheme and 50% probability ellipsoids.

The title thio­phene-chalcone derivative contains a planar oxa­diazole, a thio­phene and two phenyl rings (Fig. 1 ▸). The phenyl [*A* (C1–C6) and *D* (C16–C21)], oxa­diazole [*B* (O1/N1/N2/C7/C8)], and thio­phene [*C* (S2/C12–C15)] rings are oriented at dihedral angles of *A*/*B* = 22.61 (10)°, *A*/*C* = 49.16 (10)°, *A*/*D* = 51.78 (9)°, *B*/*C* = 27.45 (10)°, *B*/*D* = 67.07 (8)° and *C*/*D* = 87.22 (8)°. The chlorine atom Cl1 is displaced by −0.0389 (11) Å from the plane of the phenyl ring *A*. Atoms C22 and C9 are only −0.003 (4) Å and −0.002 (3) Å away from phenyl ring *D*. Atoms S1 and C9 are −0.0433 (9) Å and 0.002 (3) Å distant from the oxa­diazole, *B*, ring plane, while atom C11 resides 0.016 (3) Å above the thio­phene, *C*, ring plane. The specified atoms are, hence, essentially coplanar with the planes of the rings they are attached to. The bond lengths (Allen *et al.*, 1987 ▸) and angles are all within normal ranges.

## 3. Supra­molecular features

**Figure 2:**
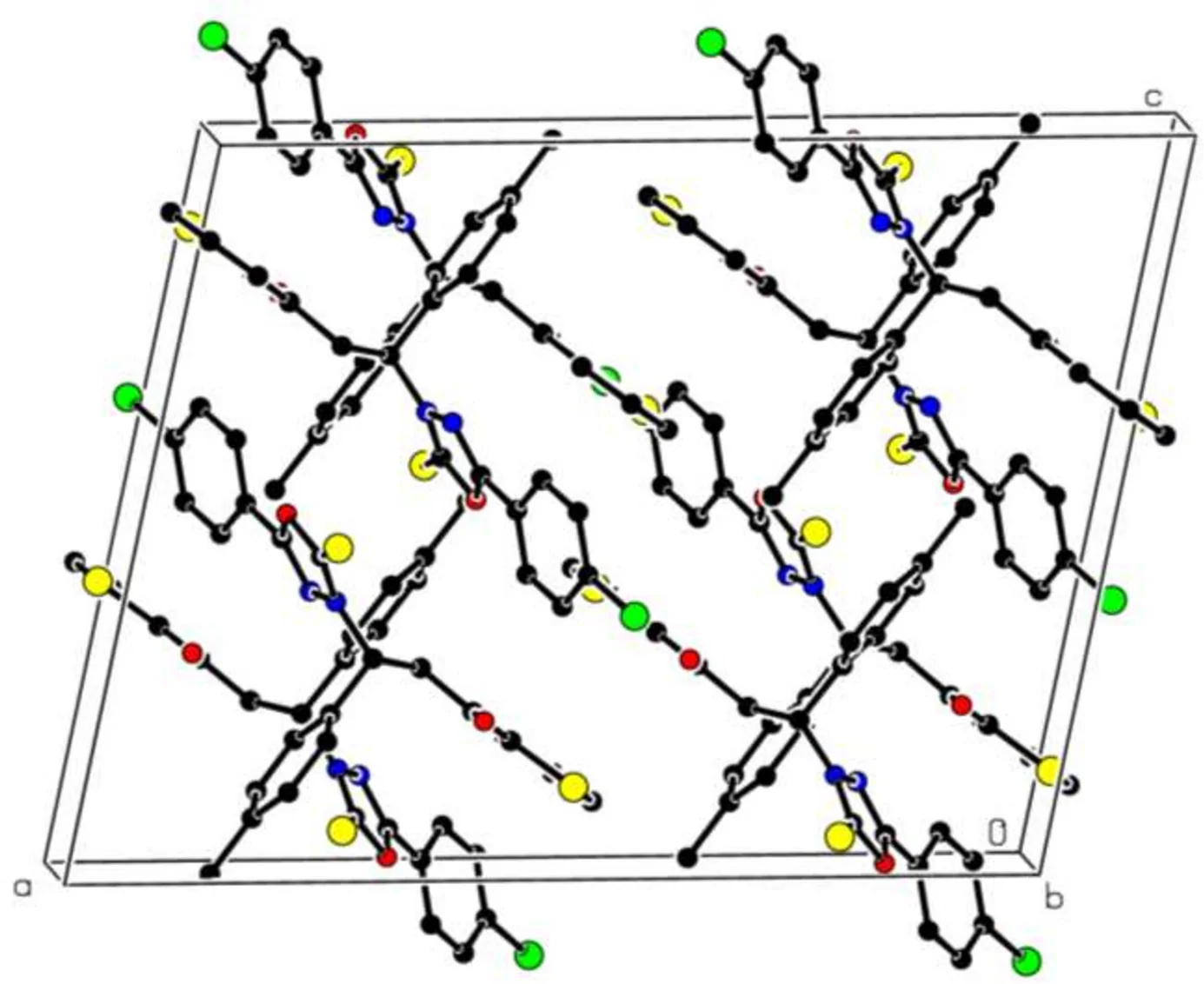
A partial packing diagram viewed down the *b*–axis direction.

In the crystal, the thio­phene-to-thio­phene contacts form pairs, which are arranged along the *c*-axis direction, and adjacent pairs are also essentially arranged in front and behind in the *a*-axis direction, albeit slightly displaced with regard to the *b*-axis direction. The chloro­phenyl-to-chloro­phenyl contacts also form pairs with contacts arranged along the *a*-axis direction. However, none of these pairs has further contacts that would extend into columns, ribbons or layers (Fig. 2 ▸). The π–π stacking inter­actions involve parallel chloro­phenyl rings *A* and approximately parallel thio­phene rings *C*. The centroid-to-centroid distances are 3.6612 (18) Å [dihedral angle = 0.00 (16)° slippage = 1.466 Å] and 3.844 (3) Å [dihedral angle = 2.0 (2)°; slippage = 0.871 Å], respectively. Together with the two C—H⋯π(ring) inter­actions (Table 1 ▸), these contacts help consolidate the overall packing.

**Figure 3:**
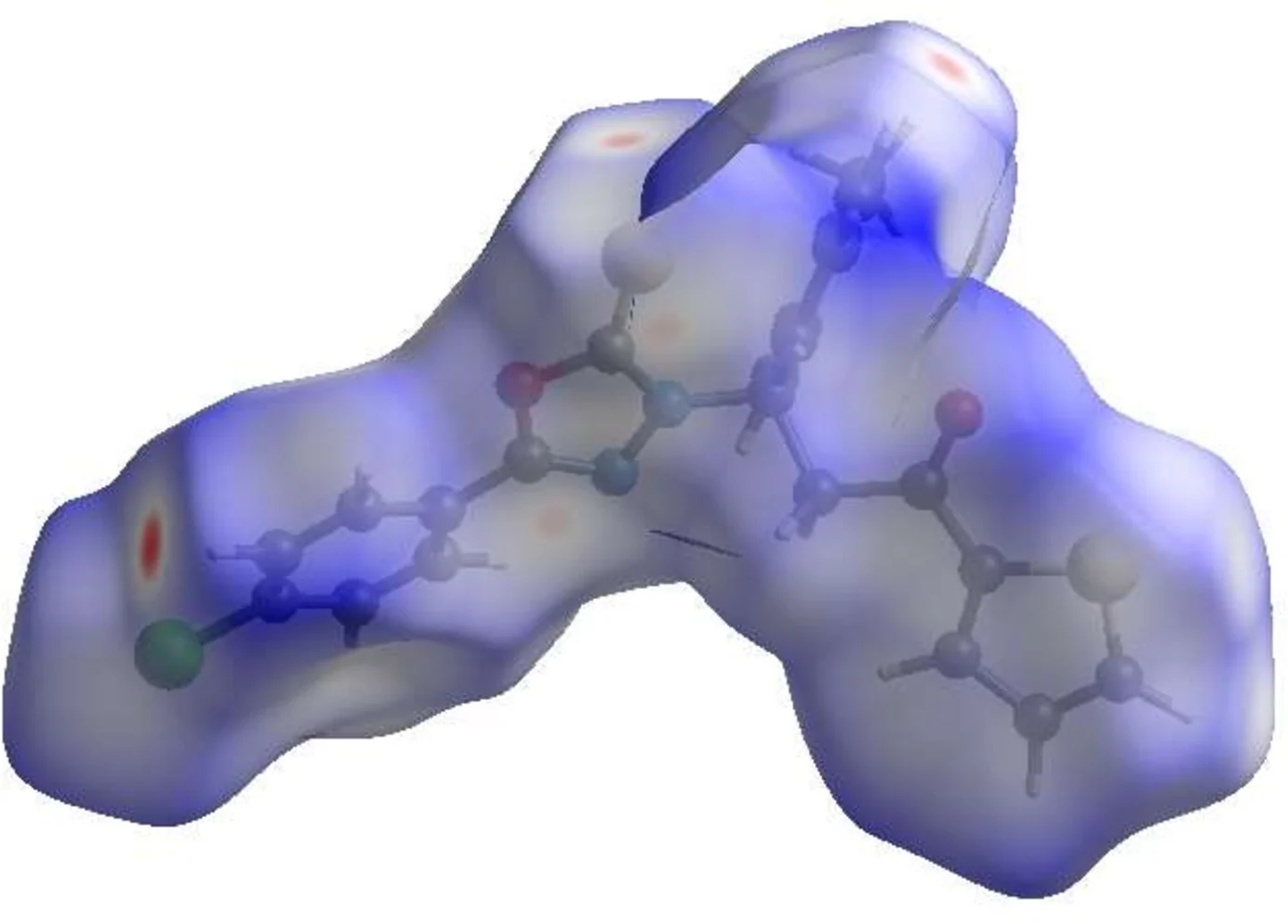
View of the three-dimensional Hirshfeld surface for the title mol­ecule plotted over *d*_norm_ in the range from −0.2794 to 1.6393 a.u.

**Figure 4:**
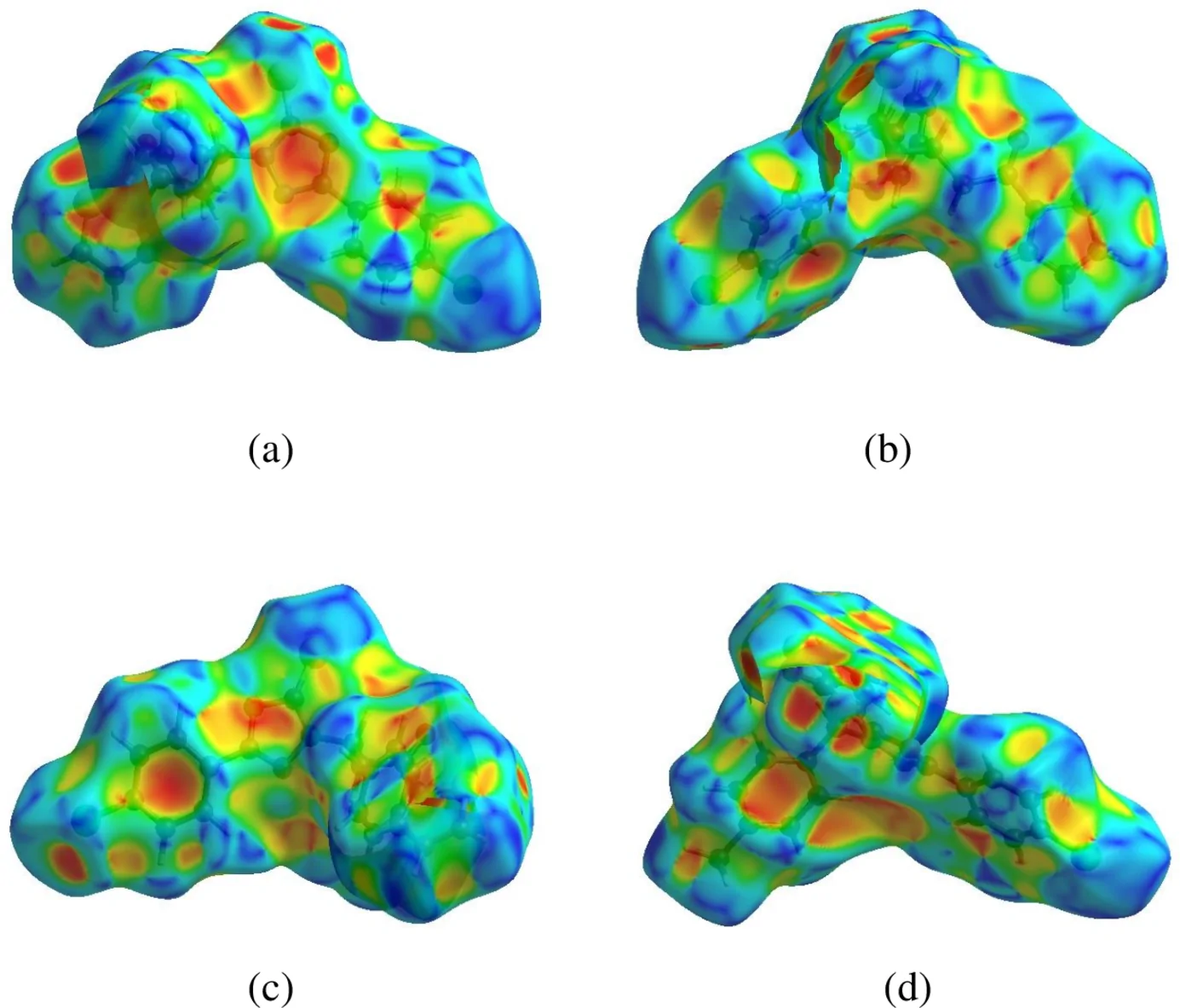
Four orientations of the shape-index showing (*a*) and (*b*) the π–π and (*c*) and (*d*) the C—H⋯π(ring) inter­actions.

The inter­molecular inter­actions in the crystal were further visualized through a Hirshfeld surface (HS) analysis using *CrystalExplorer* 17.5 (Spackman *et al.*, 2021 ▸). Fig. 3 ▸ shows the Hirshfeld surface for the title mol­ecule plotted over *d*_norm_. The white surface indicates contacts with distances equal to the sum of van der Waals radii, and the red and blue colours indicate distances shorter and longer than the van der Waals radii, respectively. The π–π stackings and the two C—H⋯π(ring) inter­actions are indicated in Fig. 4 ▸*a*,*b* by the presence of adjacent red and blue triangles, and Fig. 4 ▸*c*,*d* by the presence of red π-holes, respectively.

**Figure 5:**
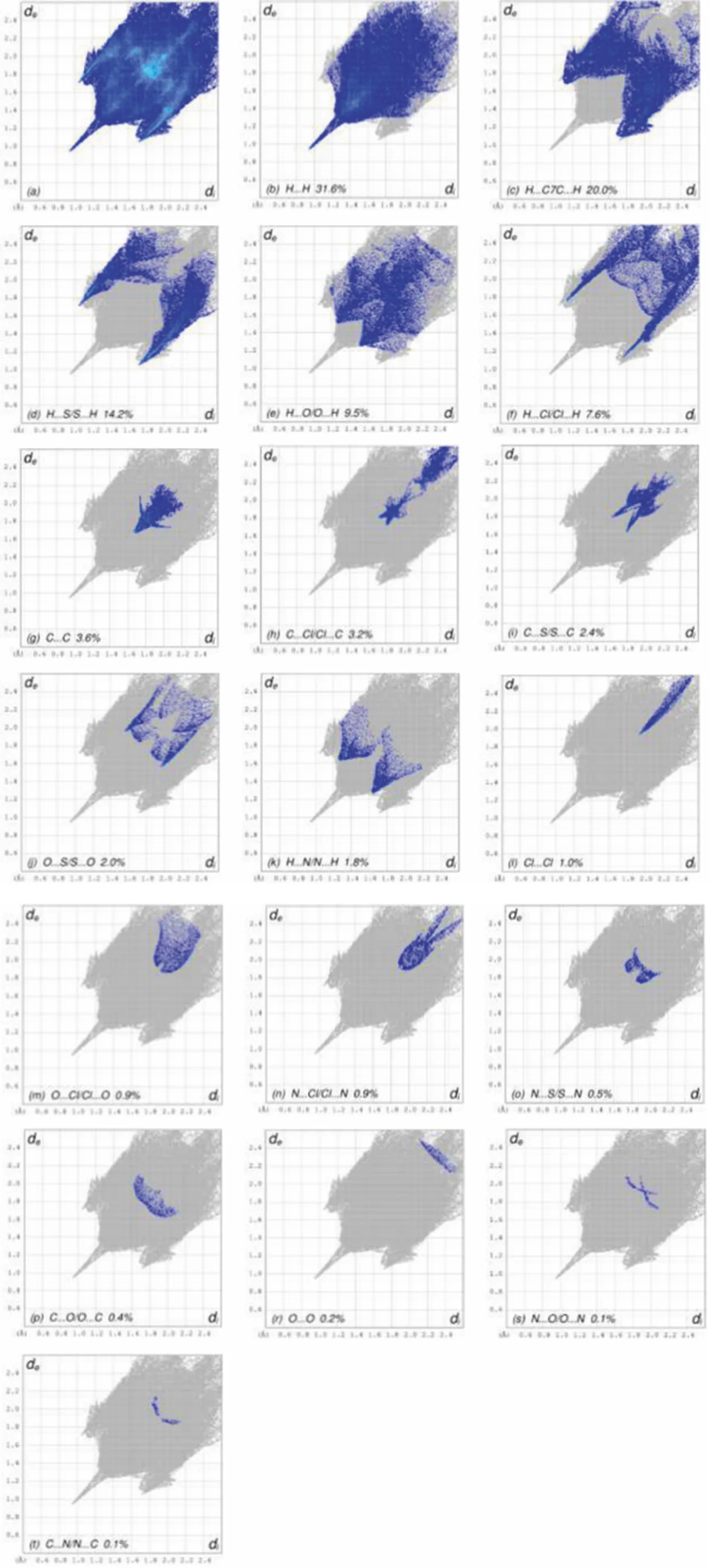
The full two-dimensional fingerprint plots for the title mol­ecule, showing (*a*) all inter­actions, and delineated into (*b*) H⋯H, (*c*) H⋯C/C⋯H, (*d*) H⋯ S/S⋯H, (*e*) H⋯O/O⋯H, (*f*) H⋯Cl/Cl⋯H, (*g*) C⋯C, (*h*) C⋯Cl/Cl⋯C, (*i*) C⋯S/S⋯C, (*j*) O⋯S/S⋯O, (*k*) H⋯N/N⋯H, (*l*) Cl⋯Cl, (*m*) O⋯Cl/Cl⋯O, (*n*) N⋯Cl/Cl⋯N, (*o*) N⋯S/S⋯N, (*p*) C⋯O/O⋯C, (*r*) O⋯O, (*s*) N⋯O/O⋯N and (*t*) C⋯N/N⋯ C inter­actions. The *d*_i_ and *d*_e_ values are the closest inter­nal and external distances (in Å) from given points on the Hirshfeld surface.

The overall two-dimensional fingerprint plot is shown in Fig. 5 ▸*a* and those delineated into H⋯H, H⋯C/C⋯H, H⋯S/S⋯H, H⋯O/O⋯H, H⋯Cl/Cl⋯H, C⋯C, C⋯Cl/Cl⋯C, C⋯S/S⋯C, O⋯S/S⋯O, H⋯N/N⋯H, Cl⋯Cl, O⋯Cl/Cl⋯O, N⋯Cl/Cl⋯N, N⋯S/S⋯N, C⋯O/O⋯C, O⋯O, N⋯O/O⋯N and C⋯N/N⋯C inter­actions are illustrated in Fig. 5 ▸(*b*)–(*t*), respectively. According to the two-dimensional fingerprint plots the H⋯H, H⋯C/C⋯ H, H⋯S/S⋯H and H⋯O/O⋯H contacts make the most significant contributions to the Hirshfeld surface, at 31.6%, 20.0%, 14.2% and 9.5%, respectively (Fig. 5 ▸).

## 4. Database survey

A search of the Cambridge Structural Database (CSD, Version 6.01, updated February 2026; Groom *et al.*, 2016 ▸) revealed that the crystal structure of the title compound has not been reported previously. However, with the exclusion of metal containing structures, ten structurally related compounds containing both thio­phene and 1,3,4-oxa­diazole-2(3*H*)-thione moieties were identified. These are *N*-[2-(2-thien­yl)-1-(5-thioxo-4,5-di­hydro-1,3,4-oxa­diazol-2-yl)vin­yl]benzamide (CSD refcode ENOPEE; Sharma *et al.*, 2016 ▸), 5-(2-thien­yl)-1,3,4-oxa­diazole-2(3*H*)-thione (FUSDII; Burcu Arslan *et al.*, 2014 ▸), *N*-[1-(5-sulfanyl­idene-4,5-di­hydro-1,3,4-oxa­diazol-2-yl)-2-(2-thien­yl)vin­yl]acetamide (HAJHAF; Karanth *et al.*, 2019 ▸), 4-methyl-*N*-[1-(5-sulfanyl­idene-4,5-di­hydro-1,3,4-oxa­diazol-2-yl)-2-(thio­phen-2-yl)ethen­yl]benzamide (HAJHOT; Karanth *et al.*, 2019 ▸), 3-[(4-phenyl­piperazin-1-yl)meth­yl]-5-(2-thien­yl)-1,3,4-oxa­diazole-2(3*H*)-thione (IDOBUA; El-Emam *et al.*, 2013 ▸), 3-[(4-phenyl­piperidin-1-yl)meth­yl]-5-(thio­phen-2-yl)-1,3,4-oxa­diazole-2(3*H*)-thione (MURSAX; Al-Wahaibi *et al.*, 2025 ▸), 3-{[4-(4-fluoro­phen­yl)piperazin-1-yl]meth­yl}-5-(2-thien­yl)-1,3,4-oxa­diazole-2(3*H*)-thione (MUXBAK; Al-Omary *et al.*, 2015*a* ▸), 3-{[4-(2-meth­oxy­phen­yl)piperazin-1-yl]meth­yl}-5-(2-thien­yl)-1,3,4-oxa­dia­zole-2(3*H*)-thione (NUZJUP; Al-Alshaikh *et al.*, 2016 ▸), 3-[(4-benzyl­piperazin-1-yl)meth­yl]-5-(2-thien­yl)-1,3,4-oxa­diazole-2(3*H*)-thione (VUBYUO; Al-Omary *et al.*, 2015*b* ▸), and 3-{[meth­yl(phen­yl)amino]­meth­yl}-5-(2-thien­yl)-1,3,4-oxa­dia­zole-2(3*H*)-thione (ZAPJIL; El-Emam *et al.*, 2012 ▸). Notably, in only three of these structures are the thio­phene and oxa­diazole moieties not directly linked to each other (ENOPEE, HAJHAF, HAJHOT). Two of these mol­ecules have only the two planar ring systems which comprised the search criteria (FUSDII and HAJHAF), the other eight all have a single additional phenyl ring while none consists of four planar ring systems as the title compound does.

Further thio­phene-bearing mol­ecules with structural features resembling those of the title compound include (*E*)-1-(2-amino­phen­yl)-3-(thio­phen-2-yl)prop-2-en-1-one (AFAXUC; Chantrapromma *et al.*, 2013 ▸), 1-(4-amino­phen­yl)-3-(thio­phen-2-yl)prop-2-en-1-one (ROTNUN; da Silva *et al.*, 2024 ▸), 1-[3-(4-meth­oxy- phen­yl)sydnon-4-yl]-3-(thio­phen-2-yl)prop-2-en-1-one (EVAWIH; Shih, 2004 ▸), (*E*)-1-(1*H*-pyrrol-2-yl)-3-(thio­phen-2-yl) prop-2-en-1-one (RUFLEM; Sweeting *et al.*, 2020 ▸), (2*E*)-1-(4-bromo­phen­yl)-3-(thio­phen-2-yl)prop-2-en-1-one (GENXED01; Arshad *et al.*, 2017 ▸) and 1-(4-meth­oxy­phen­yl)-3-(thio­phen-2-yl)prop-2-en-1-one (ZOV­POS; Pugh *et al.*, 2019 ▸). In addition, compounds incorporating related 1,3,4-oxa­diazole frameworks were also found, including 4-hy­droxy-3-{[5-(pyridin-3-yl)-1,3,4-oxa­diazol-2-yl]sulfan­yl}pent-3-en-2-one (CARJIS; Zhang *et al.*, 2021 ▸), 5-(4-meth­oxy­phen­yl)-1,3,4-oxa­diazol-2-yl­methanone (DIPGUI; Bhukta *et al.*, 2023 ▸) and 1-(4-chloro­phen­yl)-2-{[5-(4-chloro­phen­yl)-1,3,4-oxa­diazol-2-yl]sulfan­yl}ethan-1-one (GAVMOI; Kumar *et al.*, 2017 ▸).

## 5. Synthesis and crystallization

Methyl 4-chloro­benzoate (15 ml, 0.10 mol) was refluxed with hydrazine hydrate (6 ml, 0.12 mol) in ethanol (10 ml) for 4 h. The reaction mixture was cooled and poured into ice-cold water. The resulting solid was filtered, washed with water and dried to afford 4-chloro­benzohydrazide (91%, m.p. 428 K). 4-Chloro­benzohydrazide (3.41 g, 0.02 mol) was then treated with KOH (0.02 mol) and carbon di­sulfide (0.04 mol) in ethanol under reflux for 4 h. The reaction mixture was cooled, acidified with dilute HCl, and the precipitated solid was filtered, washed with water, and recrystallized from ethanol to furnish 5-(4-chloro­phen­yl)-1,3,4-oxa­diazole-2-thiol (83%, m.p. 503 K). Subsequently, 5-(4-chloro­phen­yl)-1,3,4-oxa­diazole-2-thiol was refluxed with the (2*Z*)-3-(4-methyl­phen­yl)-1-(thio­phen-2-yl)prop-2-en-1-one in ethanol for 4 h. After completion of the reaction, the mixture was cooled and poured into ice-cold water. The precipitated product was filtered, washed thoroughly with water, dried, and recrystallized from ethanol to afford 3-[5-(4-chloro­phen­yl)-2-thioxo-1,3,4-oxa­diazol-3(2*H*)-yl]-3-(4-methyl­phen­yl)-1-(thio­phen-2-yl) propan-1-one as a pure solid in 80% yield (m.p. 433 K). IR (KBr) cm^−1^: 1645 (C=O str.), 1594 (C=N str.), 1214 (C=S str.) and 3069 (Ar. C—H str.).

## 6. Refinement

Crystal data, data collection and structure refinement details are summarized in Table 2 ▸. The C-bound hydrogen-atom positions were calculated geometrically at distances of 0.93 Å (for aromatic CH), 0.98 Å (for methine CH), 0.97 Å (for methyl­ene CH) and 0.96 Å (for methyl CH) and refined using a riding model by applying the constraint of *U*_iso_(H) = *k* × *U*_eq_(C), where *k* = 1.5 for methyl H atoms *and* k = 1.2 for the other H atoms. Four reflections, 0 2 0, −2 0 2, 0 0 2 and −1 1 2, were omitted, the first three being affected by the beam stop and the fourth being a clear outlier.

## Supplementary Material

Crystal structure: contains datablock(s) I, global. DOI: 10.1107/S2056989026008819/yz2084sup1.cif

Structure factors: contains datablock(s) I. DOI: 10.1107/S2056989026008819/yz2084Isup2.hkl

Supporting information file. DOI: 10.1107/S2056989026008819/yz2084Isup3.cml

CCDC reference: 2583252

Additional supporting information:  crystallographic information; 3D view; checkCIF report

## Figures and Tables

**Table 1:** Hydrogen-bond geometry (Å, °) *Cg*3 and *Cg*4 are the centroids of the C1–C6 and C16–C21 rings.

*D*—H⋯*A*	*D*—H	H⋯*A*	*D*⋯*A*	*D*—H⋯*A*
C20—H20⋯*Cg*3^i^	0.93	2.83	3.726 (3)	164
C22—H22*B*⋯*Cg*4^ii^	0.96	2.92	3.671 (4)	136

Symmetry codes: (i) 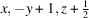; (ii) 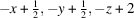.

**Table 2:** Experimental details

Crystal data	
Chemical formula	C_22_H_17_ClN_2_O_2_S_2_
*M*_r_	440.94
Crystal system, space group	Monoclinic, *C*2/*c*
Temperature (K)	300
*a*, *b*, *c* (Å)	20.7798 (3), 13.1963 (4), 16.1047 (4)
β (°)	102.440 (3)
*V* (Å^3^)	4312.49 (19)
*Z*	8
Radiation type	Mo *K*α
μ (mm^−1^)	0.39
Crystal size (mm)	0.35 × 0.25 × 0.18

Data collection	
Diffractometer	Bruker D8 Venture Diffractometer
No. of measured, independent and observed [*I* > 2σ(*I*)] reflections	53818, 3823, 2660
*R*_int_	0.117
(sin θ/λ)_max_ (Å^−1^)	0.597

Refinement	
*R*[*F*^2^ > 2σ(*F*^2^)], *wR*(*F*^2^), *S*	0.051, 0.133, 1.06
No. of reflections	3823
No. of parameters	263
H-atom treatment	H-atom parameters constrained
Δρ_max_, Δρ_min_ (e Å^−3^)	0.21, −0.34

Computer programs: *APEX4*, *SAINT* and *XPREP* (Bruker, 2021 ▸), *SHELXT2018/2* (Sheldrick, 2015*a* ▸), *SHELXL2018/3* (Sheldrick, 2015*b* ▸) and *ORTEP-3 for Windows* and *WinGX* publication routines (Farrugia, 2012 ▸).

## supplementary crystallographic information

### 3-[5-(4-Chlorophenyl)-2-sulfanylidene-1,3,4-oxadiazol-3-yl]-3-(4-methylphenyl)-1-(thiophen-2-yl)propan-1-one Crystal data

**Table:**

C_22_H_17_ClN_2_O_2_S_2_	*F*(000) = 1824
*M**_r_* = 440.94	*D*_x_ = 1.358 Mg m^−^^3^
Monoclinic, *C*2/*c*	Mo *K*α radiation, λ = 0.71073 Å
*a* = 20.7798 (3) Å	Cell parameters from 9893 reflections
*b* = 13.1963 (4) Å	θ = 2.9–24.4°
*c* = 16.1047 (4) Å	µ = 0.39 mm^−^^1^
β = 102.440 (3)°	*T* = 300 K
*V* = 4312.49 (19) Å^3^	Block, colourless
*Z* = 8	0.35 × 0.25 × 0.18 mm

### 3-[5-(4-Chlorophenyl)-2-sulfanylidene-1,3,4-oxadiazol-3-yl]-3-(4-methylphenyl)-1-(thiophen-2-yl)propan-1-one Data collection

**Table:**

Bruker D8 Venture Diffractometer	*R*_int_ = 0.117
φ and ω scans	θ_max_ = 25.1°, θ_min_ = 3.4°
53818 measured reflections	*h* = −24→24
3823 independent reflections	*k* = −15→15
2660 reflections with *I* > 2σ(*I*)	*l* = −19→19

### 3-[5-(4-Chlorophenyl)-2-sulfanylidene-1,3,4-oxadiazol-3-yl]-3-(4-methylphenyl)-1-(thiophen-2-yl)propan-1-one Refinement

**Table:**

Refinement on *F*^2^	0 restraints
Least-squares matrix: full	Hydrogen site location: inferred from neighbouring sites
*R*[*F*^2^ > 2σ(*F*^2^)] = 0.051	H-atom parameters constrained
*wR*(*F*^2^) = 0.133	*w* = 1/[σ^2^(*F*_o_^2^) + (0.0485*P*)^2^ + 5.5114*P*] where *P* = (*F*_o_^2^ + 2*F*_c_^2^)/3
*S* = 1.06	(Δ/σ)_max_ < 0.001
3823 reflections	Δρ_max_ = 0.21 e Å^−^^3^
263 parameters	Δρ_min_ = −0.34 e Å^−^^3^

### 3-[5-(4-Chlorophenyl)-2-sulfanylidene-1,3,4-oxadiazol-3-yl]-3-(4-methylphenyl)-1-(thiophen-2-yl)propan-1-one Special details

**Table:**

Geometry. All esds (except the esd in the dihedral angle between two l.s. planes) are estimated using the full covariance matrix. The cell esds are taken into account individually in the estimation of esds in distances, angles and torsion angles; correlations between esds in cell parameters are only used when they are defined by crystal symmetry. An approximate (isotropic) treatment of cell esds is used for estimating esds involving l.s. planes.

### 3-[5-(4-Chlorophenyl)-2-sulfanylidene-1,3,4-oxadiazol-3-yl]-3-(4-methylphenyl)-1-(thiophen-2-yl)propan-1-one Fractional atomic coordinates and isotropic or equivalent isotropic displacement parameters (Å^2^)

**Table:**

	*x*	*y*	*z*	*U*_iso_*/*U*_eq_	
Cl1	−0.02304 (5)	0.68488 (8)	0.36140 (7)	0.0940 (4)	
S1	0.21099 (4)	0.09611 (6)	0.55083 (6)	0.0646 (3)	
S2	0.51040 (4)	0.33068 (8)	0.88521 (6)	0.0716 (3)	
O1	0.15497 (9)	0.27611 (14)	0.50923 (12)	0.0492 (5)	
O2	0.40086 (11)	0.20490 (18)	0.79037 (17)	0.0766 (7)	
N1	0.19842 (11)	0.37647 (17)	0.61720 (14)	0.0444 (6)	
N2	0.22459 (11)	0.27964 (17)	0.62889 (14)	0.0433 (6)	
C1	0.03143 (15)	0.5946 (3)	0.4151 (2)	0.0606 (9)	
C2	0.04901 (17)	0.5143 (3)	0.3706 (2)	0.0698 (10)	
H2	0.032596	0.509239	0.312306	0.084*	
C3	0.09082 (16)	0.4415 (3)	0.41243 (19)	0.0586 (8)	
H3	0.102859	0.386888	0.382601	0.070*	
C4	0.11520 (13)	0.4497 (2)	0.49971 (18)	0.0459 (7)	
C5	0.09805 (14)	0.5322 (2)	0.54302 (19)	0.0497 (7)	
H5	0.115268	0.538910	0.601040	0.060*	
C6	0.05581 (15)	0.6043 (2)	0.5010 (2)	0.0548 (8)	
H6	0.043768	0.659303	0.530460	0.066*	
C7	0.15780 (13)	0.3708 (2)	0.54492 (17)	0.0435 (7)	
C8	0.19816 (14)	0.2172 (2)	0.56537 (18)	0.0460 (7)	
C9	0.27418 (13)	0.2523 (2)	0.70549 (17)	0.0437 (7)	
H9	0.294754	0.188761	0.693418	0.052*	
C10	0.32768 (14)	0.3332 (2)	0.72144 (19)	0.0506 (7)	
H10A	0.337773	0.353284	0.667709	0.061*	
H10B	0.311617	0.392485	0.746326	0.061*	
C11	0.38986 (14)	0.2951 (2)	0.78047 (19)	0.0508 (7)	
C12	0.43695 (14)	0.3706 (2)	0.82279 (19)	0.0526 (8)	
C13	0.43258 (19)	0.4740 (3)	0.8214 (3)	0.0882 (13)	
H13	0.396509	0.510482	0.791966	0.106*	
C14	0.4906 (3)	0.5181 (4)	0.8709 (4)	0.126 (2)	
H14	0.497143	0.587718	0.877001	0.151*	
C15	0.5353 (2)	0.4492 (3)	0.9083 (3)	0.0953 (14)	
H15	0.575662	0.465943	0.943140	0.114*	
C16	0.24206 (13)	0.2332 (2)	0.77969 (17)	0.0424 (6)	
C17	0.24210 (15)	0.1367 (2)	0.81390 (19)	0.0488 (7)	
H17	0.262005	0.083791	0.790687	0.059*	
C18	0.21309 (15)	0.1179 (2)	0.8818 (2)	0.0542 (8)	
H18	0.213774	0.052413	0.903324	0.065*	
C19	0.18314 (14)	0.1939 (2)	0.91841 (19)	0.0498 (7)	
C20	0.18410 (17)	0.2903 (2)	0.8847 (2)	0.0639 (9)	
H20	0.164964	0.343337	0.908721	0.077*	
C21	0.21262 (17)	0.3099 (2)	0.8164 (2)	0.0597 (8)	
H21	0.211976	0.375448	0.795043	0.072*	
C22	0.15170 (17)	0.1746 (3)	0.9930 (2)	0.0653 (9)	
H22A	0.104615	0.174914	0.974141	0.098*	
H22B	0.164729	0.226663	1.034900	0.098*	
H22C	0.165739	0.109833	1.017591	0.098*	

### 3-[5-(4-Chlorophenyl)-2-sulfanylidene-1,3,4-oxadiazol-3-yl]-3-(4-methylphenyl)-1-(thiophen-2-yl)propan-1-one Atomic displacement parameters (Å^2^)

**Table:**

	*U*^11^	*U*^22^	*U*^33^	*U*^12^	*U*^13^	*U*^23^
Cl1	0.0765 (7)	0.0946 (7)	0.1055 (8)	0.0220 (6)	0.0074 (6)	0.0429 (6)
S1	0.0683 (6)	0.0478 (5)	0.0757 (6)	−0.0032 (4)	0.0109 (5)	−0.0161 (4)
S2	0.0460 (5)	0.0927 (7)	0.0693 (6)	−0.0034 (4)	−0.0026 (4)	0.0089 (5)
O1	0.0486 (12)	0.0504 (12)	0.0448 (11)	−0.0060 (9)	0.0013 (9)	−0.0091 (9)
O2	0.0548 (14)	0.0589 (15)	0.105 (2)	0.0017 (11)	−0.0062 (13)	0.0120 (14)
N1	0.0457 (13)	0.0436 (13)	0.0407 (13)	−0.0005 (11)	0.0023 (11)	−0.0026 (10)
N2	0.0441 (13)	0.0417 (13)	0.0417 (13)	−0.0007 (10)	0.0044 (11)	−0.0039 (10)
C1	0.0454 (18)	0.063 (2)	0.071 (2)	0.0015 (15)	0.0070 (16)	0.0187 (18)
C2	0.066 (2)	0.088 (3)	0.0461 (19)	0.006 (2)	−0.0075 (17)	0.0044 (18)
C3	0.058 (2)	0.070 (2)	0.0438 (18)	0.0051 (16)	0.0020 (15)	−0.0057 (15)
C4	0.0380 (16)	0.0526 (17)	0.0444 (17)	−0.0045 (13)	0.0032 (13)	−0.0002 (13)
C5	0.0446 (17)	0.0534 (18)	0.0477 (17)	−0.0074 (14)	0.0022 (14)	−0.0024 (14)
C6	0.0496 (18)	0.0501 (18)	0.066 (2)	−0.0033 (14)	0.0149 (16)	0.0018 (16)
C7	0.0385 (15)	0.0487 (16)	0.0418 (16)	−0.0037 (13)	0.0057 (13)	−0.0057 (13)
C8	0.0398 (16)	0.0513 (17)	0.0473 (17)	−0.0056 (13)	0.0102 (13)	−0.0028 (14)
C9	0.0413 (16)	0.0424 (15)	0.0451 (16)	0.0017 (12)	0.0040 (13)	0.0005 (13)
C10	0.0411 (16)	0.0544 (17)	0.0534 (18)	−0.0026 (13)	0.0036 (14)	0.0057 (14)
C11	0.0439 (17)	0.0567 (19)	0.0519 (18)	0.0007 (14)	0.0102 (14)	0.0055 (15)
C12	0.0431 (17)	0.064 (2)	0.0477 (17)	−0.0010 (14)	0.0028 (14)	−0.0016 (15)
C13	0.074 (3)	0.064 (2)	0.107 (3)	−0.0009 (19)	−0.025 (2)	−0.016 (2)
C14	0.102 (4)	0.076 (3)	0.167 (5)	−0.008 (3)	−0.040 (3)	−0.038 (3)
C15	0.064 (3)	0.106 (3)	0.099 (3)	−0.012 (2)	−0.017 (2)	−0.017 (3)
C16	0.0370 (15)	0.0421 (15)	0.0457 (16)	0.0013 (12)	0.0036 (12)	−0.0011 (13)
C17	0.0560 (18)	0.0391 (15)	0.0517 (18)	0.0030 (13)	0.0122 (15)	−0.0035 (13)
C18	0.064 (2)	0.0408 (16)	0.0592 (19)	−0.0011 (14)	0.0158 (16)	0.0079 (14)
C19	0.0435 (16)	0.0520 (17)	0.0546 (18)	0.0028 (14)	0.0121 (14)	0.0045 (14)
C20	0.071 (2)	0.0532 (19)	0.075 (2)	0.0200 (16)	0.0325 (19)	0.0060 (17)
C21	0.072 (2)	0.0445 (17)	0.068 (2)	0.0130 (16)	0.0276 (18)	0.0105 (16)
C22	0.066 (2)	0.067 (2)	0.069 (2)	0.0035 (17)	0.0272 (18)	0.0094 (17)

### 3-[5-(4-Chlorophenyl)-2-sulfanylidene-1,3,4-oxadiazol-3-yl]-3-(4-methylphenyl)-1-(thiophen-2-yl)propan-1-one Geometric parameters (Å, º)

**Table:**

Cl1—C1	1.740 (3)	C10—C11	1.516 (4)
S1—C8	1.644 (3)	C10—H10A	0.9700
S2—C15	1.664 (4)	C10—H10B	0.9700
S2—C12	1.719 (3)	C11—C12	1.458 (4)
O1—C8	1.371 (3)	C12—C13	1.368 (5)
O1—C7	1.371 (3)	C13—C14	1.420 (5)
O2—C11	1.216 (3)	C13—H13	0.9300
N1—C7	1.284 (3)	C14—C15	1.345 (6)
N1—N2	1.386 (3)	C14—H14	0.9300
N2—C8	1.336 (3)	C15—H15	0.9300
N2—C9	1.472 (3)	C16—C21	1.381 (4)
C1—C2	1.373 (5)	C16—C17	1.387 (4)
C1—C6	1.373 (5)	C17—C18	1.380 (4)
C2—C3	1.370 (4)	C17—H17	0.9300
C2—H2	0.9300	C18—C19	1.378 (4)
C3—C4	1.392 (4)	C18—H18	0.9300
C3—H3	0.9300	C19—C20	1.384 (4)
C4—C5	1.381 (4)	C19—C22	1.508 (4)
C4—C7	1.457 (4)	C20—C21	1.381 (4)
C5—C6	1.370 (4)	C20—H20	0.9300
C5—H5	0.9300	C21—H21	0.9300
C6—H6	0.9300	C22—H22A	0.9600
C9—C16	1.510 (4)	C22—H22B	0.9600
C9—C10	1.523 (4)	C22—H22C	0.9600
C9—H9	0.9800		
			
C15—S2—C12	92.10 (19)	H10A—C10—H10B	108.0
C8—O1—C7	106.1 (2)	O2—C11—C12	121.3 (3)
C7—N1—N2	103.4 (2)	O2—C11—C10	121.2 (3)
C8—N2—N1	112.4 (2)	C12—C11—C10	117.5 (3)
C8—N2—C9	126.1 (2)	C13—C12—C11	129.7 (3)
N1—N2—C9	121.5 (2)	C13—C12—S2	111.3 (2)
C2—C1—C6	121.1 (3)	C11—C12—S2	119.1 (2)
C2—C1—Cl1	119.1 (3)	C12—C13—C14	110.8 (4)
C6—C1—Cl1	119.8 (3)	C12—C13—H13	124.6
C3—C2—C1	119.8 (3)	C14—C13—H13	124.6
C3—C2—H2	120.1	C15—C14—C13	113.2 (4)
C1—C2—H2	120.1	C15—C14—H14	123.4
C2—C3—C4	119.7 (3)	C13—C14—H14	123.4
C2—C3—H3	120.1	C14—C15—S2	112.6 (3)
C4—C3—H3	120.1	C14—C15—H15	123.7
C5—C4—C3	119.6 (3)	S2—C15—H15	123.7
C5—C4—C7	120.4 (3)	C21—C16—C17	117.7 (3)
C3—C4—C7	119.9 (3)	C21—C16—C9	121.9 (3)
C6—C5—C4	120.4 (3)	C17—C16—C9	120.4 (2)
C6—C5—H5	119.8	C18—C17—C16	121.1 (3)
C4—C5—H5	119.8	C18—C17—H17	119.4
C5—C6—C1	119.4 (3)	C16—C17—H17	119.4
C5—C6—H6	120.3	C19—C18—C17	121.5 (3)
C1—C6—H6	120.3	C19—C18—H18	119.2
N1—C7—O1	113.2 (2)	C17—C18—H18	119.2
N1—C7—C4	128.4 (3)	C18—C19—C20	117.0 (3)
O1—C7—C4	118.4 (2)	C18—C19—C22	122.1 (3)
N2—C8—O1	104.9 (2)	C20—C19—C22	120.9 (3)
N2—C8—S1	131.0 (2)	C21—C20—C19	122.0 (3)
O1—C8—S1	124.1 (2)	C21—C20—H20	119.0
N2—C9—C16	110.8 (2)	C19—C20—H20	119.0
N2—C9—C10	108.9 (2)	C16—C21—C20	120.6 (3)
C16—C9—C10	114.5 (2)	C16—C21—H21	119.7
N2—C9—H9	107.4	C20—C21—H21	119.7
C16—C9—H9	107.4	C19—C22—H22A	109.5
C10—C9—H9	107.4	C19—C22—H22B	109.5
C11—C10—C9	111.5 (2)	H22A—C22—H22B	109.5
C11—C10—H10A	109.3	C19—C22—H22C	109.5
C9—C10—H10A	109.3	H22A—C22—H22C	109.5
C11—C10—H10B	109.3	H22B—C22—H22C	109.5
C9—C10—H10B	109.3		
			
C7—N1—N2—C8	−1.9 (3)	N2—C9—C10—C11	−161.9 (2)
C7—N1—N2—C9	−179.9 (2)	C16—C9—C10—C11	73.4 (3)
C6—C1—C2—C3	0.9 (5)	C9—C10—C11—O2	20.9 (4)
Cl1—C1—C2—C3	−178.7 (3)	C9—C10—C11—C12	−161.0 (3)
C1—C2—C3—C4	−0.1 (5)	O2—C11—C12—C13	−178.0 (4)
C2—C3—C4—C5	−1.3 (5)	C10—C11—C12—C13	3.9 (5)
C2—C3—C4—C7	177.8 (3)	O2—C11—C12—S2	2.0 (4)
C3—C4—C5—C6	1.8 (4)	C10—C11—C12—S2	−176.1 (2)
C7—C4—C5—C6	−177.3 (3)	C15—S2—C12—C13	−0.6 (3)
C4—C5—C6—C1	−1.0 (4)	C15—S2—C12—C11	179.4 (3)
C2—C1—C6—C5	−0.4 (5)	C11—C12—C13—C14	−179.1 (4)
Cl1—C1—C6—C5	179.2 (2)	S2—C12—C13—C14	0.9 (5)
N2—N1—C7—O1	1.0 (3)	C12—C13—C14—C15	−1.0 (7)
N2—N1—C7—C4	179.4 (3)	C13—C14—C15—S2	0.6 (7)
C8—O1—C7—N1	0.2 (3)	C12—S2—C15—C14	0.0 (4)
C8—O1—C7—C4	−178.5 (2)	N2—C9—C16—C21	−67.6 (3)
C5—C4—C7—N1	−21.6 (5)	C10—C9—C16—C21	56.1 (4)
C3—C4—C7—N1	159.2 (3)	N2—C9—C16—C17	113.2 (3)
C5—C4—C7—O1	156.8 (3)	C10—C9—C16—C17	−123.1 (3)
C3—C4—C7—O1	−22.4 (4)	C21—C16—C17—C18	0.6 (4)
N1—N2—C8—O1	2.0 (3)	C9—C16—C17—C18	179.9 (3)
C9—N2—C8—O1	179.9 (2)	C16—C17—C18—C19	−0.2 (5)
N1—N2—C8—S1	−178.3 (2)	C17—C18—C19—C20	−0.7 (5)
C9—N2—C8—S1	−0.4 (4)	C17—C18—C19—C22	−179.5 (3)
C7—O1—C8—N2	−1.3 (3)	C18—C19—C20—C21	1.1 (5)
C7—O1—C8—S1	179.0 (2)	C22—C19—C20—C21	180.0 (3)
C8—N2—C9—C16	−98.7 (3)	C17—C16—C21—C20	−0.3 (5)
N1—N2—C9—C16	79.0 (3)	C9—C16—C21—C20	−179.5 (3)
C8—N2—C9—C10	134.5 (3)	C19—C20—C21—C16	−0.7 (5)
N1—N2—C9—C10	−47.8 (3)		

### 3-[5-(4-Chlorophenyl)-2-sulfanylidene-1,3,4-oxadiazol-3-yl]-3-(4-methylphenyl)-1-(thiophen-2-yl)propan-1-one Hydrogen-bond geometry (Å, º)

*Cg*3 and *Cg*4 are the centroids of the C1–C6 and C16–C21 rings.

**Table:**

*D*—H···*A*	*D*—H	H···*A*	*D*···*A*	*D*—H···*A*
C20—H20···Cg3^i^	0.93	2.83	3.726 (3)	164
C22—H22*B*···Cg4^ii^	0.96	2.92	3.671 (4)	136

Symmetry codes: (i) *x*, −*y*+1, *z*+1/2; (ii) −*x*+1/2, −*y*+1/2, −*z*+2.
